# Spiders did not repeatedly gain, but repeatedly lost, foraging webs

**DOI:** 10.7717/peerj.6703

**Published:** 2019-04-04

**Authors:** Jonathan A. Coddington, Ingi Agnarsson, Chris A. Hamilton, Jason E. Bond

**Affiliations:** 1 Department of Entomology, National Museum of Natural History, Smithsonian Institution, Washington, D.C., USA; 2 Department of Biology, University of Vermont, Burlington, VT, United States of America; 3 Department of Entomology, Plant Pathology, & Nematology, University of Idaho, Moscow, ID, United States of America; 4 Department of Entomology and Nematology, University of California, Davis, Davis, CA, United States of America

**Keywords:** Spider webs, Orb webs, Homology, Character optimization, Phylogenomics, Araneae, Silk, Spider

## Abstract

Much genomic-scale, especially transcriptomic, data on spider phylogeny has accumulated in the last few years. These data have recently been used to investigate the diverse architectures and the origin of spider webs, concluding that the ancestral spider spun no foraging web, that spider webs evolved *de novo* 10–14 times, and that the orb web evolved at least three times. These findings in fact result from a particular phylogenetic character coding strategy, specifically coding the *absence* of webs as logically equivalent, and homologous to, 10 other observable (i.e., not absent) web architectures. “Absence” of webs should be regarded as inapplicable data. To be analyzed properly by character optimization algorithms, it should be coded as “?” because these codes—or their equivalent—are handled differently by such algorithms. Additional problems include critical misspellings of taxon names from one analysis to the next (misspellings cause some optimization algorithms to drop terminals, which affects taxon sampling and results), and mistakes in spider natural history. In sum, the method causes character optimization algorithms to produce counter-intuitive results, and does not distinguish absence from secondary loss. Proper treatment of missing entries and corrected data instead imply that foraging webs are primitive for spiders and that webs have been lost ∼5–7 times, not gained 10–14 times. The orb web, specifically, may be homologous (originated only once) although lost 2–6 times.

## Introduction

“Not all living spiders spin webs, but since 1950 web-building species have been found in almost all the families of spiders once thought of as wandering hunters. It now seems likely that all spiders who actively hunt their prey, or use little or no silk in prey capture, are descendants of web builders.” Shear (1994).

The evolution of silk use and web architectures, in particular the origin, modification, and/or loss of the orb web is one of the more fundamental questions in spider biology. Although the ancestral spider has always been presumed to employ silk in prey capture, modern spiders do spin two rather different kinds of orb webs whose homology is hotly debated: the Uloboridae and Deinopidae with mechanically adhesive micro-threads produced by the cribellum—a special spinning plate—and the araneoids with viscid glue produced by two pairs of specialized silk spigots. In the decades preceding the 1980’s, arachnologists generally hypothesized that the cribellate and viscid silk orb weavers were reciprocally monophyletic and only distantly related; under this scenario the two kinds of orb webs were convergent.

Lehtinen (1967) and Forster (1970) most prominently argued that the cribellum was primitive for araneomorph spiders, and that ecribellate lineages, including araneoids, evolved from cribellate ancestors. If true, this hypothesis refutes the best argument for orb web convergence and orb weaver polyphyly. Orb weavers—the Orbiculariae—were arguably monophyletic, (reviewed in Coddington, 1986b; Coddington & Levi, 1991; Miller et al., 2010). This ‘single origin’ hypothesis found evidence in silk gland and spigot morphology as well as observations of similar web-building behaviors—often identical stereotypical means of laying down similar threads (Eberhard, 1982; Coddington, 1986a; Coddington, 1986b; Coddington, 1986c; reviewed in Eberhard, 1990). Skeptics countered that web architecture was strongly selected, that the orb itself was adaptive and likely convergent, and that morphological and behavioral similarities could be explained as convergence (e.g., Kovoor & Peters, 1988).

Although the behavioral and morphological evidence seemed compatible with monophyly, molecular evidence repeatedly questioned orb weaver monophyly. Early targeted gene analyses were largely discounted due to sparse taxon sampling and the perceived inadequacy of the genes sampled (e.g., rRNA and mtDNA genes, Agnarsson, Coddington & Kuntner, 2013). However, Bond et al. (2014) and Fernández, Hormiga & Giribet (2014) assembled massive phylogenomic datasets that clearly refuted orbicularian monophyly. Both placed the cribellate orb weavers close to the RTA clade, which includes a vast number of cursorial, non-web building taxa, like jumping and wolf spiders.

If Orbiculariae was not monophyletic, could the two sorts of orb webs nevertheless be homologous? Phylogenomic data analyzed by Bond et al. (2014) and Garrison et al. (2016) supported homology, but the orb evolved earlier than previously supposed and was lost several times (the ancient origin hypothesis). Comparative and functional morphology (spinneret spigots and silk glands) and behavior (stereotypical motor patterns) analyzed under maximum likelihood support the ancient origin hypothesis. In the viscid orbweavers (Araneoidea), the novel aggregate (gluey) silk is placed on flagelliform fibers to produce the sticky spiral of the orb. An ortholog of the araneoid flagelliform spidroin, however, has also been found in the cribellate orb weavers, that bear so-called ‘pseudoflagelliform’ silk glands (Garb et al., 2006). Recent genomic studies suggest that the aggregate silk gene is most closely related to flagelliform spidroins (Babb et al., 2017; Collin et al., 2018). The origin of flagelliform spidroins in cribellate orb weavers, and the likely subsequent origin of aggregate spidroins in Araneoidea, derived from flagelliform spidroins further supports the idea that the transformation from cribellate to aggregate adhesives occurred in an orb weaving ancestor who already produced flagelliform silk.

Fernández et al. (2018, hereafter F&al) recently analyzed new transcriptomic data to add important taxa to the phylogenomic spider tree (Bond et al., 2014; Fernández, Hormiga & Giribet, 2014; Garrison et al., 2016). First published in April 2018, with data in a repository, the authors issued an erratum that (1) modified their homology hypothesis on web variation, (2) altered their conclusions on web evolution, (3) changed the original data in the repository, and (4) resulted in a markedly different interpretation of the data.

Their June corrected data and publication confirmed orbicularian polyphyly. Their homology hypothesis for web architectures refuted the single origin hypothesis (Blackledge et al., 2009; Bond et al., 2014; Garrison et al., 2016). They found that orb webs and their associated behaviors and machinery evolved 3–6 times.

More surprisingly, prey capture webs evolved approximately 14 times across spiders from a webless ancestor. These are bold claims of convergent evolution—and a stark departure from most other modern studies.

When closely examined these inferences of convergence, either of webs in general or orbs, derive more from what we regard as inappropriate phylogenetic methods than from the data (although we do dispute some facts, see below). All character optimization algorithms assume that the digits representing character states code for observable, real phenomena. The only exception is missing or inapplicable data, both by convention coded as “?”. F&al, however, coded absence of webs using digits (in fact, states “6” and “8”), and, given the taxon sample, “no foraging web” optimizes as the ancestral spider condition. But “absence” of webs is not the presence of anything. Absence codes in the data matrix could mean missing, if the taxon is thought to exhibit an (unknown) character state, or inapplicable if it is known to lack one, as in this case. Optimization algorithms purposely treat “?” as a special case, different from digits. Given how optimization algorithms work, coding web absence as an observed state (a digit) rather than “?” affects the results.

To disentangle the effects of this practice, we attempt to duplicate the results of F&al to investigate the effect of this methodological choice (composite coding, Strong & Lipscomb, 1999). We also correct a few empirical mistakes). We reanalyze their data (and our corrected data) to assess whether web building and the orb web evolved multiple times.

The objectives of this paper are threefold. First, we justify in more detail below why coding absence as the presence of something homologous and coordinate to observable web architectures yields illogical results in this case, as well as disputing some empirical details. Second, we reanalyze the F&al emended dataset (with their altered data but methodological problems fixed) to show that webs are primitive and homologous for spiders. Finally, we show that the orb web single origin hypothesis is still reasonable and supported by data, and recommend future improvements to homology hypotheses on web evolution.

Properly analyzed, the evidence suggests that prey capture webs are an ancient trait of all spiders. They did not independently evolve 10–14 times. Orb webs may be homologous as orb webs.

## Materials & Methods

We first attempted to replicate F&al’s results for their transcriptomic data using their corrected web codings. Their original web character was (0) orb; (1) brush sheet; (2) irregular aerial sheet; (3) irregular ground sheet; (4) stereotyped aerial sheet; (5) cobweb; (6) no foraging web; (7) aerial (above ground) silk tube; (8) tubular silk-lined burrows; (9) irregular tangle (not sheet-like). The corrected dataset changed 14% (23 of 159 for the transcriptomic matrix) of character states for webs, changed the meaning of state 8 to “no foraging silk-lined burrows,” and added an 11th state, (10) terminal line (see Table S1).

Our emended data set (Table S1) recodes 58 F&al “no foraging web” terminals as “?”. To capture the homology of all spider webs as webs, we include an additional character “webs: present; absent,” a method known as reductive coding (Strong & Lipscomb, 1999). We also take issue with an additional 27 codings that we think are factually wrong (Table S1), but generally accepted F&al’s re-codings (e.g., adding an 11th state “terminal line” to code *Segestria*) in order to test fairly the effect of reductive versus composite coding (Strong & Lipscomb, 1999). Most of these changes do not affect our two main points: webs are ancestral for spiders and orb webs may be homologous. Examples are that *Hypochilus* spins a “stereotyped aerial sheet,” not an “irregular ground sheet,” *Scytodes* spins an “irregular aerial sheet,” not “no foraging web,” and *Cicurina* and *Cepheia* spin ‘irregular ground sheet’ webs, not ‘irregular aerial sheets. Examples that do affect the optimization of webs as ancestral for spiders are *Microhexura, Porrhothele, Macrothele*, and *Megahexura* as brushed sheet webs (J Bond, pers. obs., 2001–2018), rather than “irregular ground sheet.” *Promyrmekiaphila,* scored by F&al as having a “brush sheet” builds a trapdoor and burrow (Stockman & Bond, 2008; J Bond, pers. obs., 2001–2018).

Because the tree files produced from the F&al study were not freely available, we reanalyzed the F&al preferred matrix (BUSCO_750); the preferred data matrix comprises 750 orthologous genes identified using the BUSCO pipeline (Simão et al., 2015). Using IQTree version 1.6.4 (Nguyen et al., 2015; Chernomor, Von Haeseler & Minh, 2016) we inferred a phylogeny sufficiently similar to that used by F&al to test the effect of alternative web codings. Character optimizations using the *ape* package (Paradis, Claude & Strimmer, 2004) ‘ace’ were checked against those reported by F&al to assess repeatability with our tree and their downloaded data matrix (Fig. S1). The resulting tree file is available for download with the supplemental figures and table associated with this manuscript (Supplementary Information).

We prefer to use the R package *corHMM* (Beaulieu, O’Meara & Donoghue, 2013) on an ultrametric tree to infer ancestral character states. The ‘rayDISC’ package specifically accommodates character polymorphisms and missing data. Character optimizations using equal (ER), symmetric (SYM), and all rates different (ARD) models were explored for these data using ‘rayDISC’ (*corHMM*). The *ape* package ‘ace,’ on the other hand, does not handle such data natively, but requires modification to the package code itself and in our experience often passed errors for complex character optimizations with many states and missing/inapplicable data (e.g., the ER, SYM, and ARD models failed at times). Because the results differed moderately based on the model used, AICc scores were employed to select a statistically preferred model.

## Results

We were unable to replicate exactly the online corrected results reported by F&al using the ‘ace’ character reconstruction. Although aspects of the ancestral state reconstruction in our analysis match approximately, orbs evolve four, not three times independently (Fig. S1 versus F&al Fig. 3A: Fernández et al., 2018).

**Figure 1:**
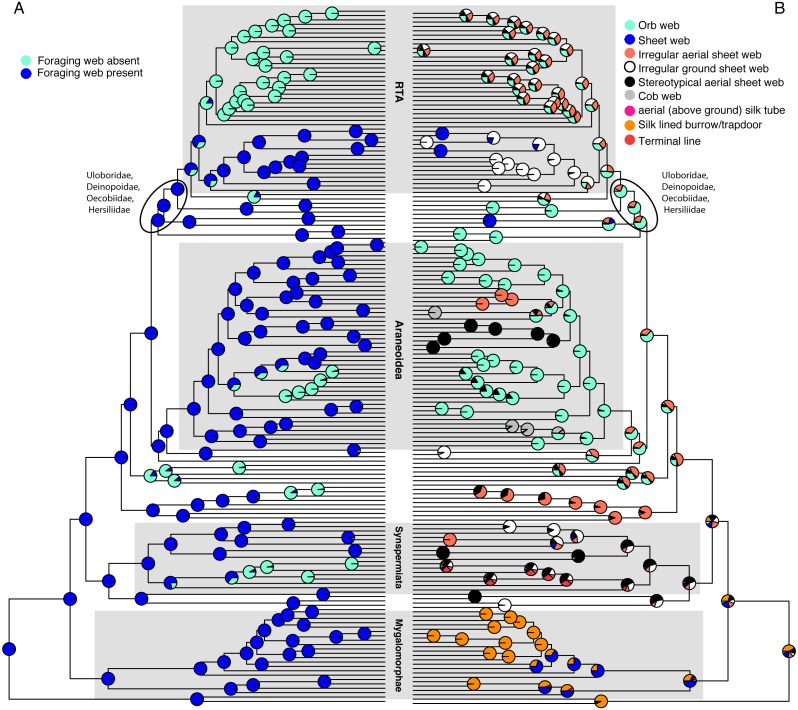
Optimizations of web presence/absence (A), and nine web architectures with web absence coded as missing data (B) under the corHMM ER model. CorHMM ER model optimization of (A) web presence/absence, showing that webs are primitive for spiders, and (B) optimization of nine web architectures with web absence coded as missing data, showing evidence for an ancient origin of the orb web.

This disparity evidently arises because their web optimization (F&al Fig. 3A: Fernández et al., 2018) includes only 158 terminals—omitting *Pararchaea*—which is present in the F&al Fig. 1A (Fernández et al., 2018) (thus 159 terminals). F&al misspelled *Pararchaea as “Pararchea.”* If taxon tree and matrix labels in ‘ace’ do not match exactly, the tip is dropped (and noted in the output file), which of course affects optimizations. F&al scored *Pararchaea* as “no foraging web” and it fell in the clade sister to tetragnathids. Rather than maintaining a larger probability of an orb web weaving ancestor (with subsequent loss further up the tree) the webless *Pararchaea* shifts the ancestral reconstruction for tetragnathids more towards a webless ancestor. As such misspellings are easy to miss in such a large tree (e.g., “*Euryops*” instead of *Euryopis*), it is possible that more of the orb web optimizations reported by F&al may be short by one additional origin (F&al Table 1: Fernández et al., 2018). Variant spelling of terminal names can affect probabilities of inferred ancestral states.

Optimizing web presence/absence on these data (Fig. 1A and Fig. S2) shows that webs are ancestral with ∼6 subsequent losses (ARD AICc = 135.7375). This optimization contradicts F&al, whose results inferred no foraging web as the ancestral spider condition. Their optimization infers that irregular aerial sheet webs derived independently three times from “no foraging web,” and brushed sheets twice. Cob webs and stereotypical aerial sheets are the only architectures clearly derived from a web building ancestor in F&al’s optimization.

“Non-foraging silk-lined burrows” is a webless condition and consequently mygalomorphs coded as such should have been scored as “no foraging web” under F&al’s corrected character state scheme. If the F&al scoring is modified to reflect that change (all taxa with state ‘8’ receive the webless score ‘6’) spiders optimize as having no foraging web plesiomorphically (i.e., spider are unequivocally primitively webless; Fig. S3, based on an ER model (AICc = 375.491).

Using the corrected character matrix (Table S1), orbs (Fig. 1B & Fig. S4 ER model, AICc = 222.8629, the ancient origin hypothesis) may have evolved once. The ancestral spider spun a web, although which architecture remains ambiguous. In general, optimized ancestral states at deep nodes are also ambiguous. The RTA clade, for example, exhibits a number of web architectures, with some probability of an orb weaving ancestor. An analysis of F&al’s matrix that includes *Pararchaea* that is only further modified by changing “no foraging web” to missing/inapplicable optimizes irregular ground and aerial sheets as the ancestral web architectures with four independent origins of the orb web (Fig. S5).

## Discussion

The most common current use of morphological characters is to map them on molecular trees rather combining them with molecules to infer phylogeny. Mapping legacy homology hypotheses on new trees risks uncritical acceptance of those hypotheses, whereas new phylogenetic topologies can require revisions of homology hypotheses (Grande & Bemis, 1998; Poe & Wiens, 2000; Rieppel & Kearney, 2002; Jenner, 2002).

Hypotheses of homology start with observations of similarity. These are the primary homology hypotheses (De Pinna, 1991) to be tested by congruence. For a meaningful phylogenetic test, a precise circumscription of a character and each of its states is needed (Hawkins, Hughes & Scotl, 1997; Freudenstein, 2005). Primary homology hypothesizes that two traits are the same by descent.

Secondary loss is a classic and pervasive problem in comparative biology (Strong & Lipscomb, 1999). Phylogeneticists therefore approach it with theoretical and methodological attention. The biggest pitfall is to code “absence” or “not X” as coordinate, equivalent, and homologous to a series of real, observable, alternative states, that are, among themselves, putatively homologous (Hennig, 1966; Wagner & Gauthier, 1999; Brower & De Pinna, 2014). When background evidence suggests that all absences represent losses, not primitive absences, this approach tests the homology of secondary losses (e.g., Blackledge et al., 2009).

When ‘absence’ conflates primitive absence and secondary loss, as F&al did (e.g., “no foraging web,” “non foraging silk lined burrow,” or “no web,”), such conflations, viewed as hypotheses of descent or homology, are illogical (De Pinna, 1991; Hawkins, Hughes & Scotl, 1997; Rieppel & Kearney, 2002). Jenner (2002: 75) identified the problem: “Since most unspecified “absence” states are optimized as plesiomorphies, the reconstructed ground patterns of stem species (nodes) on a cladogram are for many characters entirely ambiguous.” More seriously, coding unspecified absence states as homologous to observable states claims homology before it has been tested by congruence, rendering the test tautological at best.

Coding variation in spider webs is admittedly complicated. Difficult questions include defining webs as such, and, as putative adaptations, what they function to do. Common sense says that webs function to slow down prey until the predator can attack. Webs can also promote spider speed, enabling prey contact faster than otherwise. The common metric is time: slower prey and faster predators. This more expansive view of the use of silk by spiders to forage expands the discussion from debates about the homology of common or rare architectures, towards when, and how, spiders use, or do not use, silk to improve foraging success. This more inclusive theoretical context is more likely to capture the extensive variation of the use of silk by spiders in foraging, and thus may provide a more stable foundation for future theoretical and empirical work.

Pre-cladistic attempts at coding web architectures suggested implicitly homologous categories such as burrow, tube, sheet, cob, and orb webs, and a smattering of odd architectures that did not fit into any other category (Comstock, 1912; Kaston, 1964; Kullmann, 1972; reviewed and reiterated in Vollrath & Selden, 2007). Informed by phylogenetic theory, in another trend, arachnologists atomized behavior and morphology into multiple homology hypotheses (e.g., Blackledge et al. (2009) coded 47 characters pertinent to spinnerets, webs, and behaviors). Usable observations were overwhelmingly limited to orb weavers and their relatives because their behavior and morphology was patterned and lent itself to phylogenetic analysis.

Nevertheless, Blackledge et al. (2009) (and Garrison et al., 2016) attempted to leapfrog ignorance about non-orbweavers by using a phylogenetic perspective to update the earlier theory that whole web architectures could be homologized. They proposed nine states, one of which was, indeed, webless. Although potentially the same methodological error as attributed to F&al, whether that error actually results in any particular analysis in illogical findings depends on the taxon sample and the distribution of states. Insofar as two Blackledge et al. (2009) co-authors (Coddington, Agnarsson) are also coauthors here, we can affirm that coding absence of webs as a coordinate state to other architectures was carefully checked, and found to be innocuous, because all absences were secondary losses—nearly the opposite of the current example.

In retrospect Blackledge and co-authors should have used the more rigorous reductive coding. Coding absences as coordinate to other, real phenomena is probably never the best idea, and possibly always bad. We should have, as we do here, coded the presence or absence of webs as a controlling variable, with variation in web architecture coded only for taxa with webs, and webless taxa scored as inapplicable. Although the absence of this good idea did not affect the results of Blackledge et al. (2009), it strongly affects the results of F&al.

F&al emphasize that the orb web evolved three times. We show above that their own character hypothesis, including *Pararchaea*, implies four origins. Both the three or four origin results depend on coding “no foraging web” as a real, observable state (“6”) rather than as inapplicable data (“?”). When “no foraging web” is coded as inapplicable data (Fig. 1B), the ancient origin hypothesis is sustained. The webs of all extant orb weavers may be homologous as orbs.

Their more startling result is that ancestral spiders spun no webs and used no silk to improve foraging success. Although their Fig. 3A shows some probability of “non-foraging silk lined burrow,” and “irregular ground sheet,” the former is the same, scarcely disguised, methodological choice, and the latter includes factual errors.

F&al apply “non-foraging silk lined burrow” exclusively to liphistiomorphs and mygalomorphs. What does this mean? Both “no foraging web” and “non-foraging silk lined burrow,” share the notion of “non-foraging,” presumably intentionally. If, therefore, all are coded as “no foraging web,” it persists as the ancestral spider condition (see Fig. S3, ER model, AICc = 375.491).

However, we argue that mygalomorph spiders do use silk in prey capture. Most mygalomorphs build foraging webs; that is, the majority of species employ silk either in a sheet web or at a burrow entrance to detect, localize, and manipulate (see above) prey. Although the connection with prey capture is most obvious for mygalomorph sheet webs (Coyle, 1986; Coyle, Dellinger & Bennett, 1992; Bond & Coyle, 1995), trapdoor spiders employ silk at their burrow entrance and in the door that is used directly in prey detection. Moreover, many trapdoor spider species add silk lines, plant material, and tabs to their burrow entrance to detect prey (e.g., some nemesiids, actinopodids, cyrtaucheniids, antrodiaetids, and barychelids). Trapdoor covered burrows may not entangle or impede prey, but aspects of the burrow do clearly serve to enhance the sensory capacity of, and speed up the predator. Multiple mygalomorph taxa are attracted to the burrow entrance by vibrations (JE Bond, pers. obs., 2001–2018), demonstrating the role of silk in prey detection for these taxa.

Ancient uraraneid fossils, and close relatives like *Chimerarachne* (Wang et al., 2018; Esko & Selden, 2005) may have constructed sheet webs. Recent advances in mygalomorph systematics (Hedin & Bond, 2006; Bond et al., 2014; Hedin et al., 2018) place diplurid and hexathelid (all sheet web weavers) as the sister group to all other non-atypoid mygalomorphs. The uraraneid sister taxon to all spiders, coupled with extensive mygalomorph sampling, could resolve the ancestral spider web condition as a simple sheet web. Reductive coding of presence absence scoring optimizes foraging webs as the ancestral spider condition with secondary web loss.

## Conclusions

Reconstructing the evolution of spider webs remains an exciting yet unstable field of study: not only the origin and evolution of webs, as such, but the origin of the iconic orb web. Given the sensitivity of optimization algorithms to adjacent nodes, taxon sampling will always bedevil conclusions. Other factors include the optimization algorithm used (especially the proper handling of inapplicable/missing data), maximum likelihood rates of change among states, and subjective disagreements about which conceptual state to apply to which observed web architecture. That said, the notion that the single ancient origin hypothesis “crumbles under the weight of additional transcriptomic data coupled with a significantly increased taxon sampling” is premature (Fernández et al., 2018).

Spider genomics and NGS sequencing technologies may presage stable phylogenetic trees for spiders, but they are just beginning to influence fundamental questions about web construction, its underlying genetics, and the emergent phenotype of web architecture. Rather than homologizing whole web architectures, a more reductionist approach seems essential to accurately formulate homology hypotheses, and to accommodate new taxa and data. Coding whole webs should be abandoned for an approach that tests homology hypotheses at a much finer scale, based on the multiple independent characters of spider webs. For example: (1) silk use in prey capture; presence or absence of (2) ampullate, (3) piriform, (4) aggregate, (5) flagelliform and (6) cribellate silks; (7) web location and attachment points; (8) prey locomotion (such as web ‘designs’ focused towards aerial vs. pedestrian prey); (9) refugium location; (10) architectural elements (such as disordered vs. patterned, ordered or stereotypical); (11) pattern type (for example 2D vs. 3D), and more. This approach avoids arbitrary coding of whole webs as loosely defined conglomerate homology hypotheses, and could allow hypotheses of web architectures to emerge from nuclei of concordant, more objective homology hypotheses.

Regardless, our best efforts to reanalyze data on web architecture variation in spiders, including careful attention to the treatment of “absence” or inapplicable/missing data, suggests that the ancient single origin of the orb web is feasible. Orbs did not originate 3-6 times, and spider webs did not originate 14 times. Their ancestor spun a web. These results, after all, just reinforce prevailing views regarding the evolutionary history of spider webs. They do illustrate the pitfalls of disregarding long accepted rules for coding homology and mis-coding of “absence” characters, in particular. Although we do not make the claim that a multiple origin hypothesis is false, we strongly disagree with the assertion that a single origin hypothesis has been falsified.

##  Supplemental Information

10.7717/peerj.6703/supp-1 Figure S1 Ace character state reconstruction of Fernández et al., 2018 data set Preferred ’ace’ ancestral state reconstruction of web types using Fernández et al., 2018 coding scheme (Table S1) but including *Pararchaea*. Optimization correctly shows four, rather than three independent origins of the orb web.

10.7717/peerj.6703/supp-2 Figure S2 Optimization of web presence/absence Optimizing web presence/absence shows that webs are ancestral with ∼6 subsequent losses (ARD AICc = 135.7375). Corresponds to manuscript Fig. 1A but includes taxon names.

10.7717/peerj.6703/supp-3 Figure S3 Optimization of Fernández et al., 2018 data set with non foraging silk-lined burrows and no foraging webs treated as a single character state Optimization of Fernández et al., 2018 data set with non foraging silk-lined burrows and no foraging webs treated as a single character; spiders are unequivocally primitively webless under this scenario based on an ER model (AICc = 375.491).

10.7717/peerj.6703/supp-4 Figure S4 Ancestral state reconstruction of foraging webs—character states corrected and no foraging webs treated as missing/inapplicable Preferred ancestral state reconstruction of web types using a corrected character coding scheme (Figure S1, modified from Blackledge et al., 2009), the ER model in *corHMM*, and with webless taxa treated as inapplicable (-); tree modified as ultrametric; AICc = 222.8629. This hypothesis implies a single ancient origin of the orb web; spiders primitively use webs for foraging.

10.7717/peerj.6703/supp-5 Figure S5 Ancestral state reconstruction of web types using Fernandez et al. 2018 character uncorrected scoring scheme with no foraging webs scored as missing/inapplicable An analysis of F&al’s matrix using corHMM with an ER model that includes *Pararchaea. C* hanging “no foraging web” to missing/inapplicable optimizes irregular ground and aerial sheets as the ancestral web architectures with four independent origins of the orb web.

10.7717/peerj.6703/supp-6 Table S1 The F&al taxa, their April coding for web types, their changes to those codes in June, and the Coddington&Al codes for the same taxa

## Additional Information and Declarations

### Data Availability

The following information was supplied regarding data availability: The raw data is available in Table S1.

## References

[ref-1] Agnarsson I, Coddington JA, Kuntner M, Penney D (2013). Systematics: progress in the study of spider diversity and evolution. Spider research in the 21st century: trends and perspectives.

[ref-2] Babb PL, Lahens NF, Correa-Garhwal SM, Nicholson DN, Kim EJ, Hogenesch JB, Kuntner M, Higgins L, Hayashi CY, Agnarsson IIngi, Voight BF (2017). The Nephila clavipes genome highlights the diversity of spider silk genes and their complex expression. Nature Genetics.

[ref-3] Beaulieu JM, O’Meara BC, Donoghue MJ (2013). Identifying hidden rate changes in the evolution of a binary morphological character: the evolution of plant habit in campanulid angiosperms. Systematic Biology.

[ref-4] Blackledge TA, Scharff N, Coddington JA, Szüts T, Wenzel JW, Hayashi CY, Agnarsson I (2009). Reconstructing web evolution and spider diversification in the molecular era. Proceedings of the National Academy of Sciences of the United States of America.

[ref-5] Bond JE, Coyle FA (1995). Observations on the natural history of an Ummidia trapdoor spider from Costa Rica (Araneae, Ctenizidae). Journal of Arachnology.

[ref-6] Bond JE, Garrison NL, Hamilton CA, Godwin RL, Hedin M, Agnarsson I (2014). Phylogenomics resolves a spider backbone phylogeny and rejects a prevailing paradigm for orb web evolution. Current Biology.

[ref-7] Brower AVZ, De Pinna MCC (2014). About nothing. Cladistics.

[ref-8] Chernomor O, Von Haeseler A, Minh BQ (2016). Terrace aware data structure for phylogenomic inference from supermatrices. Systematic Biology.

[ref-9] Coddington JA, Shear W (1986a). The monophyletic origin of the orb web. Spiders: webs, behavior, and evolution.

[ref-10] Coddington JA (1986b). Orb webs in “non-orb weaving” ogre faced spiders (Araneae: Deinopidae): a question of genealogy. Cladistics.

[ref-11] Coddington JA (1986c). The genera of the spider family Theridiosomatidae. Smithsonian Contributions to Zoology.

[ref-12] Coddington JA, Levi HW (1991). Systematics and evolution of spiders (Araneae). Annual Review of Ecology and Systematics.

[ref-13] Collin MA, Clarke III TH, Ayoub NA, Hayashi CY (2018). Genomic perspectives of spider silk genes through target capture sequencing: Conservation of stabilization mechanisms and homologybased structural models of spidroin terminal regions. International Journal of Biological Macromolecules.

[ref-14] Comstock JH (1912). The evolution of the webs of spiders. Annals of the Entomological Society of America.

[ref-15] Coyle FA, Shear WA (1986). The role of silk in prey capture by non-araneomorph spiders. Spiders: webs, behavior and evolution.

[ref-16] Coyle FA, Dellinger RE, Bennett RG (1992). Retreat architecture and construction behaviour of an East African idiopine trapdoor spider (Araneae, Idiopidae). Bulletin of the British Arachnological Society.

[ref-17] De Pinna MCC (1991). Concepts and tests of homology in the cladistic paradigm. Cladistics.

[ref-18] Eberhard WG (1982). Behavioral characters for the higher classification of orb-weaving spiders. Evolution.

[ref-19] Eberhard WG (1990). Function and phylogeny of spider webs. Annual Review of Ecology and Systematics.

[ref-20] Esko KY, Selden PA (2005). First record of spiders from the Permian period (Araneae: Mesothelae). Bulletin of the British Arachnological Society.

[ref-21] Fernández R, Kallal RJ, Dimitrov D, Ballesteros JA, Arnedo MA, Giribet G, Hormiga G (2018). Phylogenomics, diversification dynamics, and comparative transcriptomics across the spider tree of life. Current Biology.

[ref-22] Fernández R, Hormiga G, Giribet G (2014). Phylogenomic analysis of spiders reveals nonmonophyly of orb weavers. Current Biology.

[ref-23] Forster RR (1970). The spiders of New Zealand, Part 3 (Desidae, Dictynidae, Hahniidae, Amaurobioididae, Nicodamidae). Otago Museum Bulletin.

[ref-24] Freudenstein JV (2005). Characters, states and homology. Systematic Biology.

[ref-25] Garb JE, DiMauro T, Vo V, Hayashi CY (2006). Silk genes support the single origin of orb webs. Science.

[ref-26] Garrison NL, Rodriguez J, Agnarsson I, Coddington JA, Griswold CE, Hamilton CA, Hedin M, Kocot KM, Ledford JM, Bond JE (2016). Spider phylogenomics: untangling the Spider Tree of Life. PeerJ.

[ref-27] Grande L, Bemis WE (1998). A comprehensive phylogenetic study of amiid fishes (Amiidae) based on comparative skeletal anatomy. An empirical search for interconnected patterns of natural history. Society of Vertebrate Paleontology Memoir.

[ref-28] Hawkins JA, Hughes CE, Scotl RW (1997). Primary homology assessment, characters and character states. Cladistics.

[ref-29] Hedin M, Bond JE (2006). Molecular phylogenetics of the spider infraorder Mygalomorphae using nuclear rRNA genes (18S and 28S): conflict and agreement with the current system of classification. Molecular Phylogenetics and Evolution.

[ref-30] Hedin M, Derkarabetian S, Ramírez MJ, Vink C, Bond JE (2018). Phylogenomic reclassification of the world’s most venomous spiders (Mygalomorphae, Atracinae), with implications for venom evolution. Scientific Reports.

[ref-31] Hennig W (1966). Phylogenetic systematics.

[ref-32] Jenner P (2002). Boolean logic and character state identity: Pitfalls of character coding in metazoan cladistics. Contributions to Zoology.

[ref-33] Kaston BJ (1964). The evolution of spider webs. American Zoologist.

[ref-34] Kovoor J, Peters HM (1988). The spinning apparatus of *Polenecia producta* (Araneae: Uloboridae): Structure and histochemistry. Zoomorphology.

[ref-35] Kullmann E (1972). The convergent development of orb webs in cribellate and ecribellate spiders. American Zoologists.

[ref-36] Lehtinen PT (1967). Classification of the cribellate spiders and some allied families, with notes on the evolution of the suborder Araneomorph. Annales Zoologici Fennici.

[ref-37] Miller JA, Carmichael A, Ramírez MJ, Spagna JC, Haddad CR, Rezác M, Johannesen J, Král J, Wang X-P, Griswold CE (2010). Phylogeny of entelegyne spiders: affinities of the family Penestomidae (NEW RANK), generic phylogeny of Eresidae, and asymmetric rates of change in spinning organ evolution (Araneae, Araneoidea, Entelegynae). Molecular Phylogenetics and Evolution.

[ref-38] Nguyen L-T, Schmidt HA, von Haeseler A, Minh BQ (2015). IQ-TREE: a fast and effective stochastic algorithm for estimating maximum likelihood phylogenies. Molecular Biology and Evolution.

[ref-39] Paradis E, Claude J, Strimmer K (2004). APE: analyses of phylogenetics and evolution in R language. Bioinformatics.

[ref-40] Poe D, Wiens JJ, Wiens JJ (2000). Character selection and the methodology of morphological phylogenetics. Phylogenetic analysis of morphological data.

[ref-41] Rieppel O, Kearney M (2002). Similarity. Biological Journal of the Linnean Society.

[ref-42] Shear WA (1994). Untangling the evolution of the web. American Scientist.

[ref-43] Simão FA, Waterhouse RM, Ioannidis P, Kriventseva EV, Zdobnov EM (2015). BUSCO: assessing genome assembly and annotation completeness with single-copy orthologs. Bioinformatics.

[ref-44] Stockman AK, Bond JE (2008). A taxonomic review of the trapdoor spider genus *Promyrmekiaphila* Schenkel (Araneae, Mygalomorphae, Cyrtaucheniidae, Euctenizinae). Zootaxa.

[ref-45] Strong EE, Lipscomb D (1999). Character coding and inapplicable data. Cladistics.

[ref-46] Vollrath F, Selden PA (2007). The role of behavior in the evolution of spiders, silks, and webs. Annual Review of Ecology and Systematics.

[ref-47] Wagner GP, Gauthier JA (1999). 1,2,3 = 2,3,4: a solution to the problem of the homology of the digits in the avian hand. Proceedings of the National Academy of Sciences of the United States of America.

[ref-48] Wang B, Dunlop JA, Selden PA, Garwood RJ, Shear WA, Müller P, Lei XJ (2018). Cretaceous arachnid *Chimerarachne yingi* gen. et sp. nov. illuminates spider origins. Nature Ecology & Evolution.

