# Supplementary figures and images for: Spiders did not repeatedly gain, but repeatedly lost, foraging webs
Source: PeerJ. 2019 Apr 4;7:e6703. doi: 10.7717/peerj.6703 (PMC6451839; doi:10.7717/peerj.6703)

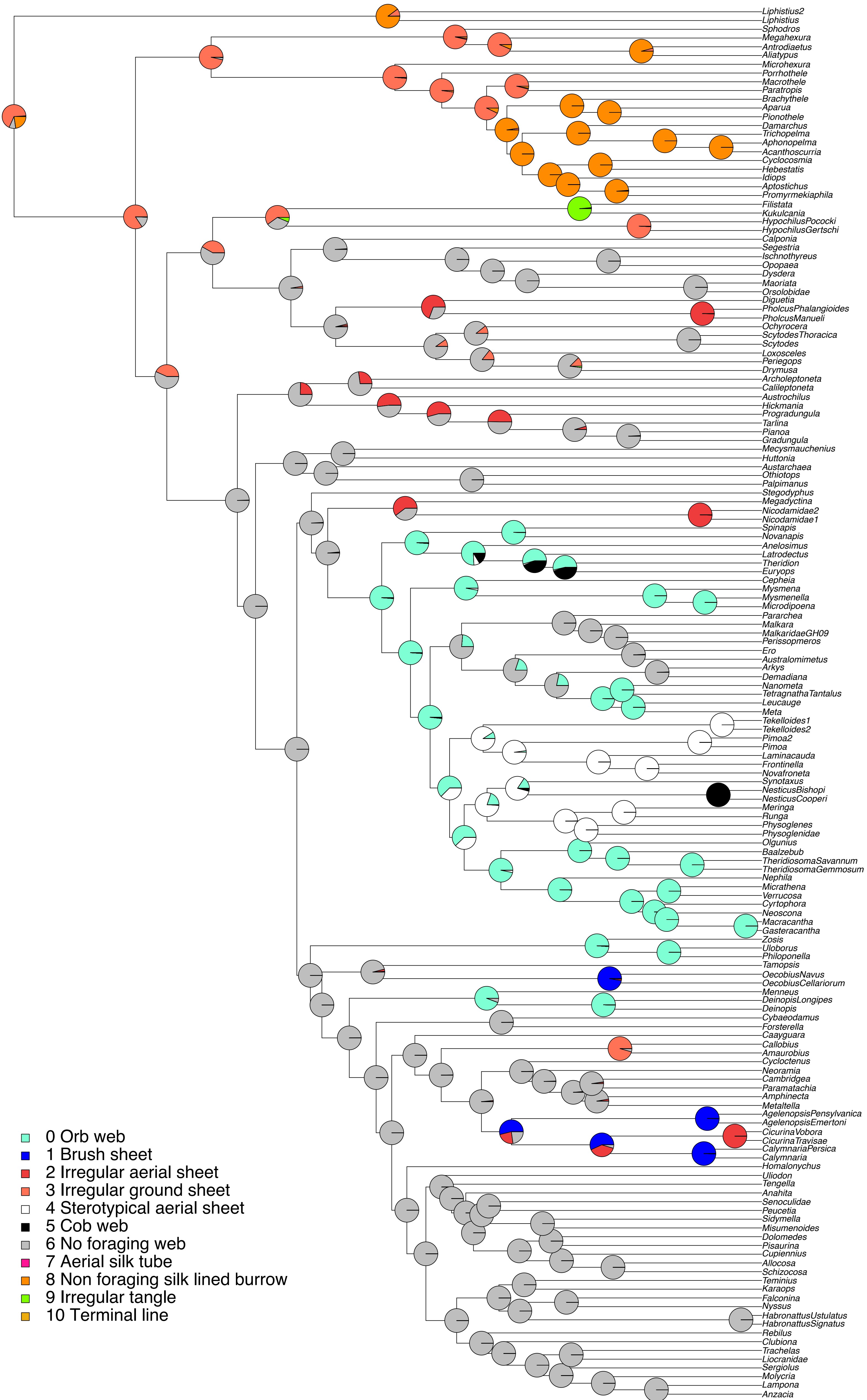

Supplement: Figure S1 — Preferred ’ace’ ancestral state reconstruction of web types using Fernández et al., 2018 coding scheme (Table S1) but including Pararchaea. Optimization correctly shows four, rather than three independent origins of the orb web. [file peerj-07-6703-s001.pdf]

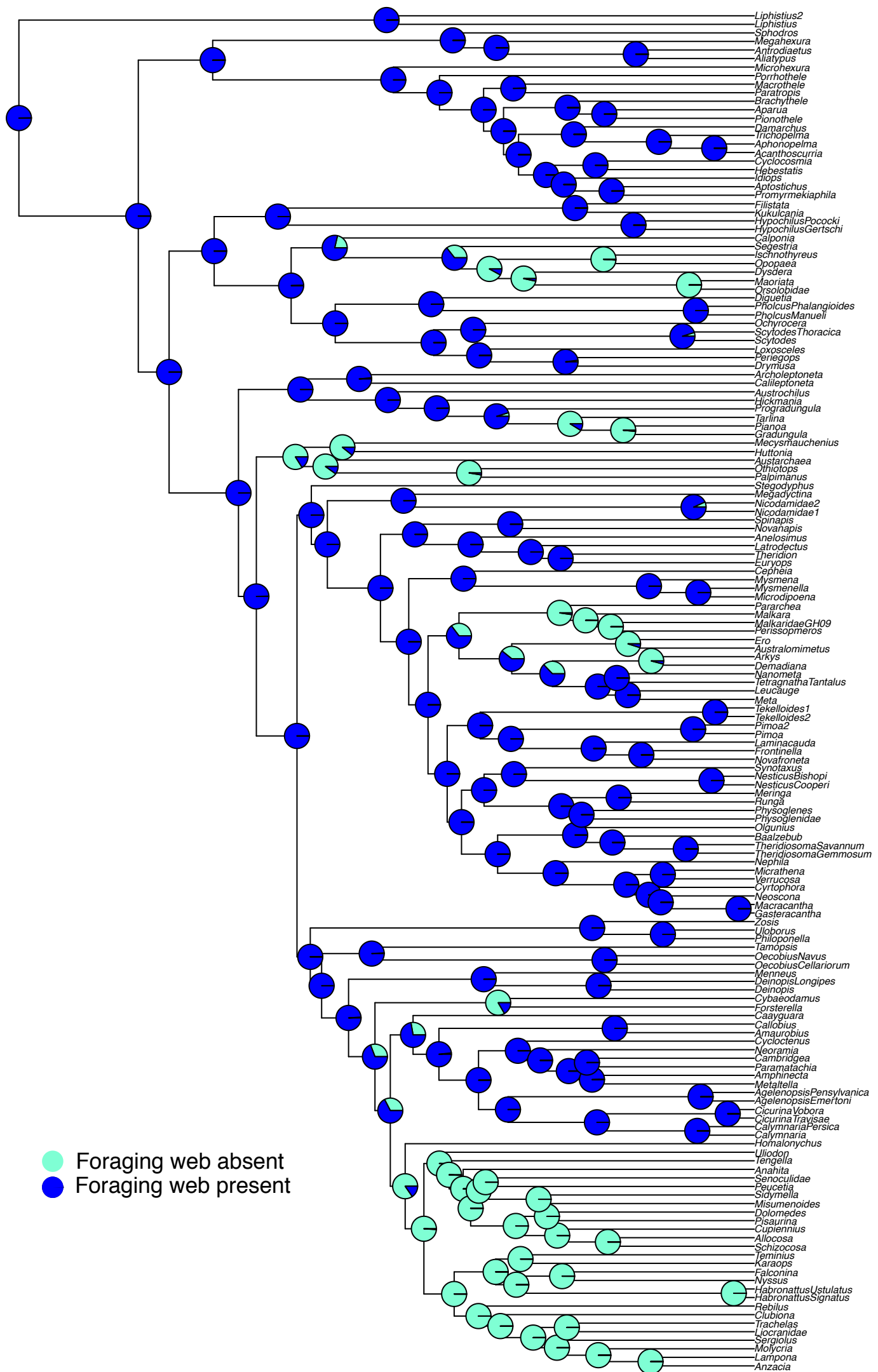

Supplement: Figure S2 — Optimizing web presence/absence shows that webs are ancestral with ∼6 subsequent losses (ARD AICc = 135.7375). Corresponds to manuscript Fig. 1A but includes taxon names. [file peerj-07-6703-s002.pdf]

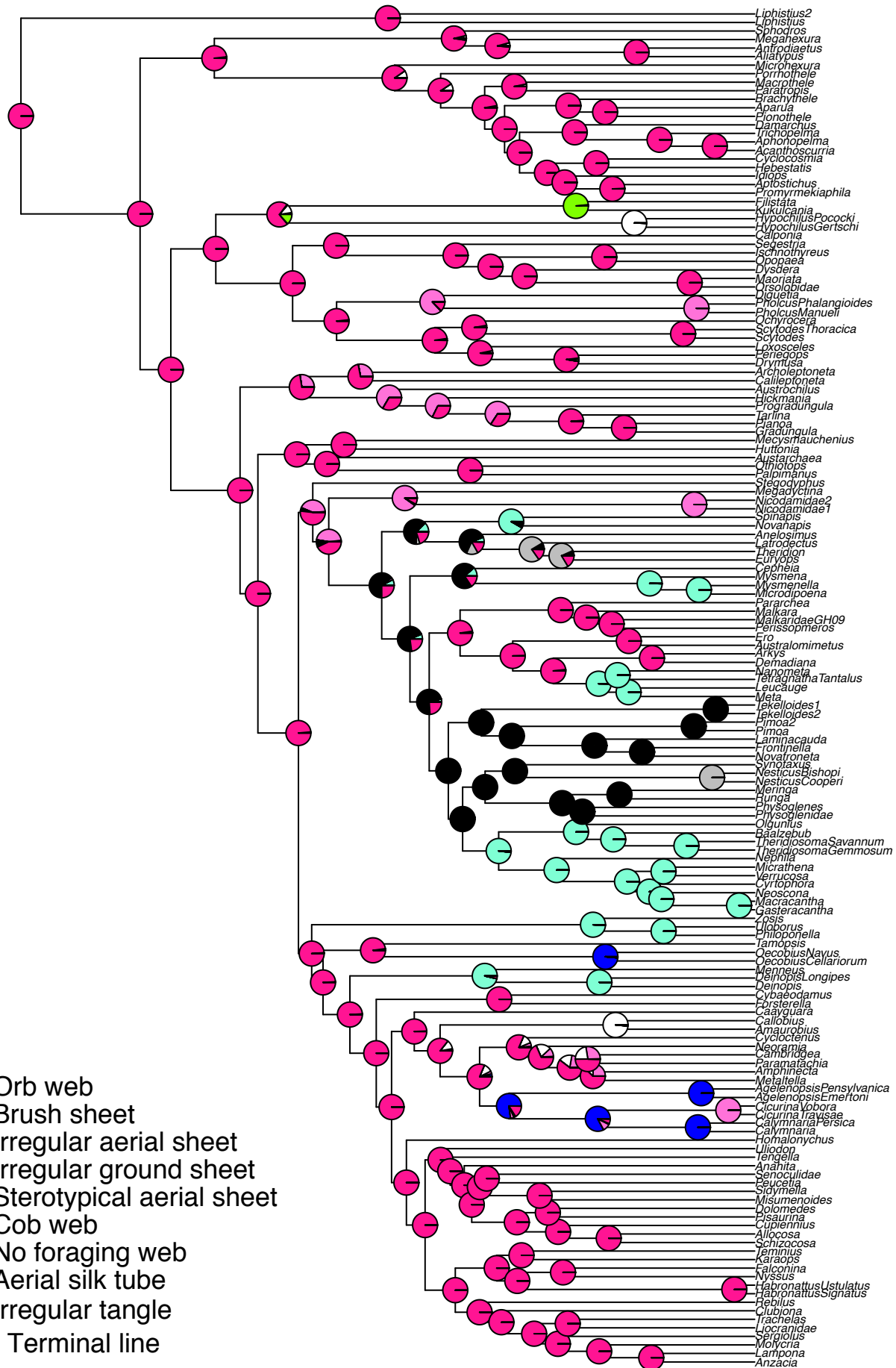

Supplement: Figure S3 — Optimization of Fernández et al., 2018 data set with non foraging silk-lined burrows and no foraging webs treated as a single character; spiders are unequivocally primitively webless under this scenario based on an ER model (AICc = 375.491). [file peerj-07-6703-s003.pdf]

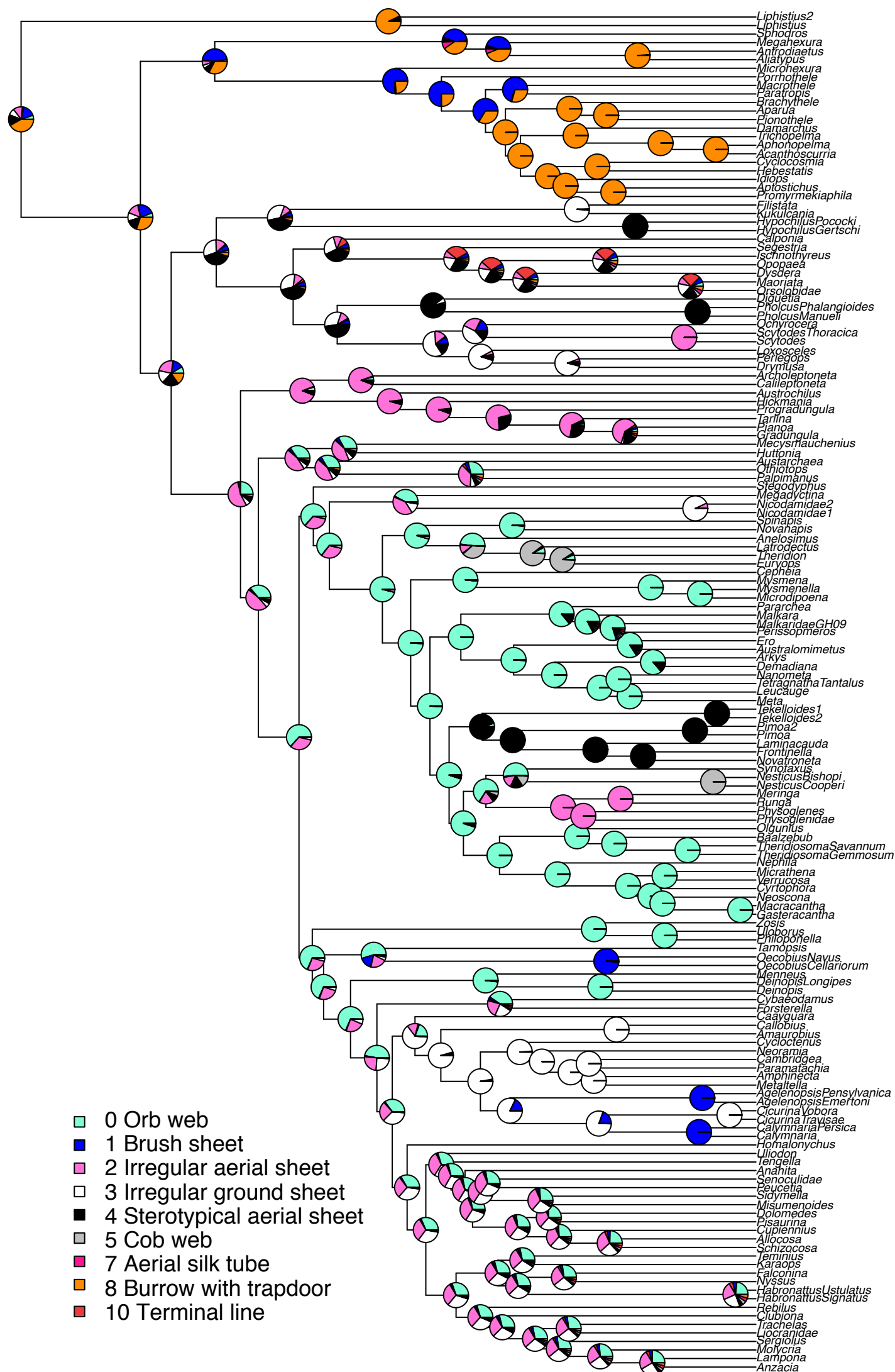

Supplement: Figure S4 — Preferred ancestral state reconstruction of web types using a corrected character coding scheme (Figure S1, modified from Blackledge et al., 2009), the ER model in corHMM, and with webless taxa treated as inapplicable (-); tree modified as ultrametric; AICc = 222.8629. This hypothesis implies a single ancient origin of the orb web; spiders primitively use webs for foraging. [file peerj-07-6703-s004.pdf]

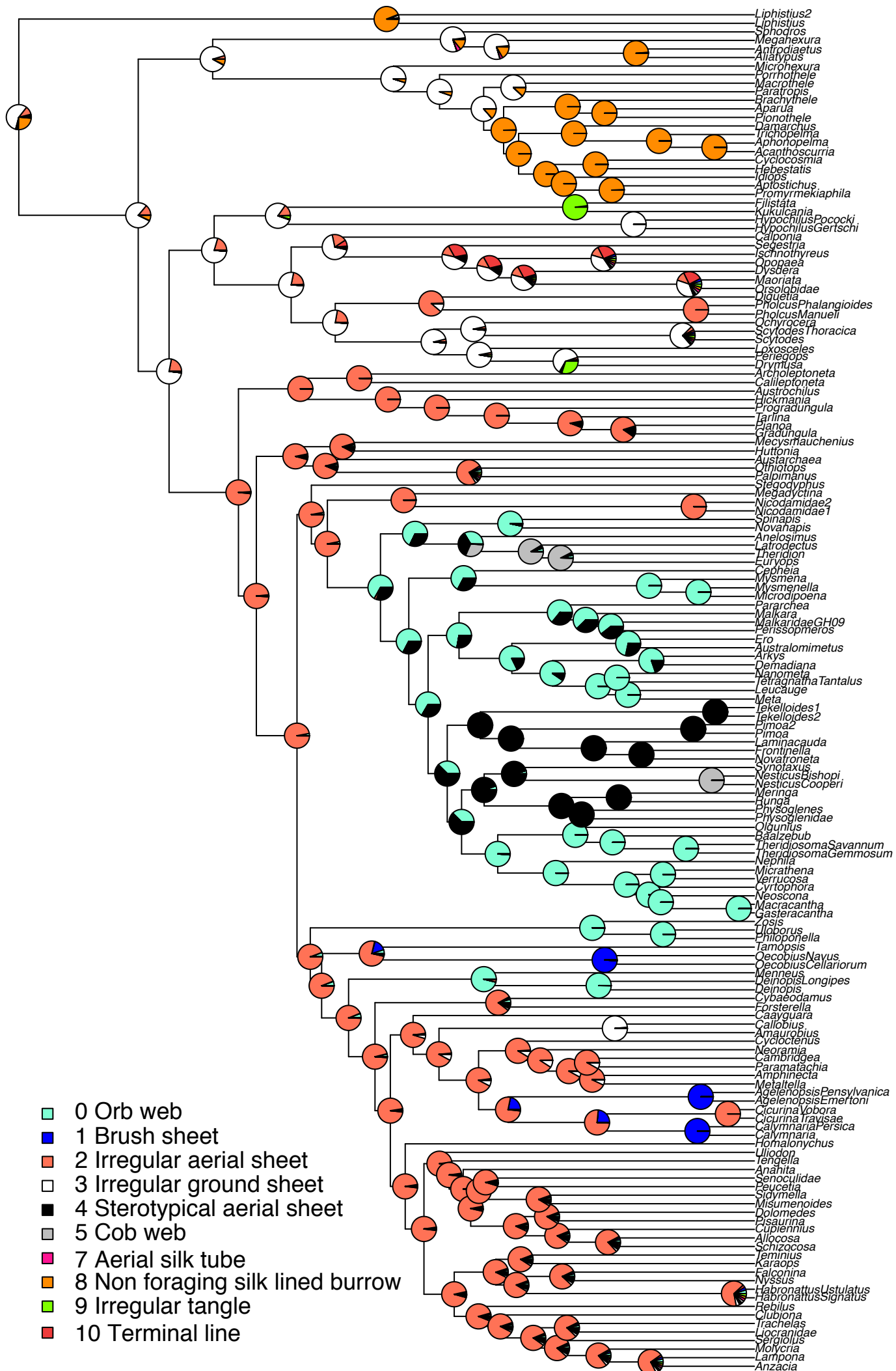

Supplement: Figure S5 — An analysis of F&al’s matrix using corHMM with an ER model that includes Pararchaea. C hanging “no foraging web” to missing/inapplicable optimizes irregular ground and aerial sheets as the ancestral web architectures with four independent origins of the orb web. [file peerj-07-6703-s005.pdf]
